# Structure–activity relationships of strigolactones via a novel, quantitative *in planta* bioassay

**DOI:** 10.1093/jxb/ery092

**Published:** 2018-03-15

**Authors:** Elena Sanchez, Emma Artuso, Chiara Lombardi, Ivan Visentin, Beatrice Lace, Wajeeha Saeed, Marco L Lolli, Piermichele Kobauri, Zahid Ali, Francesca spyrakis, Pilar Cubas, Francesca Cardinale, Cristina Prandi

**Affiliations:** 1 Centro Nacional de Biotecnología-CSIC, Plant Molecular Genetics Department, C/ Darwin, Campus UAM, Madrid, Spain; 2 Department of Chemistry, University of Turin, via P. Giuria Turin, Italy; 3 Department of Agricultural, Forestry and Food Science, Largo P. Braccini, Grugliasco (TO), Italy; 4 Department of Drug Science and Technology, University of Turin, via P. Giuria Turin, Italy; 5 Department of Biosciences, COMSATS Institute of Information Technology, Islamabad, Pakistan; 6 University of Freiburg, Faculty of Biology, Cell Biology, Schänzlestr., Freiburg, Germany

**Keywords:** Bioassay, bioisosterism, chemical space, docking, luciferase, perception, plant hormones, strigolactones, strigolactone-D-lactams

## Abstract

Strigolactones (SLs) are plant hormones with various functions in development, responses to stress, and interactions with (micro)organisms in the rhizosphere, including with seeds of parasitic plants. Their perception for hormonal functions requires an α,β-hydrolase belonging to the D14 clade in higher plants; perception of host-produced SLs by parasitic seeds relies on similar but phylogenetically distinct proteins (D14-like). D14 and D14-like proteins are peculiar receptors, because they cleave SLs before undergoing a conformational change that elicits downstream events. Structure–activity relationship data show that the butenolide D-ring is crucial for bioactivity. We applied a bioisosteric approach to the structure of SLs by synthetizing analogues and mimics of natural SLs in which the D-ring was changed from a butenolide to a lactam and then evaluating their bioactivity. This was done by using a novel bioassay based on Arabidopsis transgenic lines expressing AtD14 fused to firefly luciferase, in parallel with the quantification of germination-inducing activity on parasitic seeds. The results obtained showed that the *in planta* bioassay is robust and quantitative, and thus can be confidently added to the SL-survey toolbox. The results also showed that modification of the butenolide ring into a lactam one significantly hampers the biological activity exhibited by SLs possessing a canonical lactonic D-ring.

## Teaser

The biological activity of natural and novel strigolactone D-lactam analogues is assessed using a novel bioassay based on Arabidopsis transgenic lines expressing AtD14 fused to firefly luciferase.

## Introduction

Strigolactones (SLs) are a class of plant hormones that play several pleiotropic roles above and below ground, and whose exploitation could pave the way to innovative crop enhancement applications (Al-Babili and Bouwmeester, 2015; Cardinale *et al.*, 2018). To achieve this long-term goal, it is first necessary to fully elucidate the mechanism of action that forms the basis of SL perception by the producing plant and other organisms sensitive to SLs (Lumba *et al.*, 2017). The evolutionarily conserved SL receptor AtD14 (*Arabidopsis thaliana* DWARF14) and its orthologues in other species are members of the α,β-hydrolases superfamily (Hamiaux *et al.*, 2012). A subset of AtD14 paralogues, named AtD14-like and belonging to the same super-family, has been described as having a very similar global structure and a conserved catalytic triad (serine, histidine, and aspartate) (Zhao *et al.*, 2013). Recently, six such D14-like paralogues were characterized as SL receptors in the parasitic plant *Striga hermontica*, whose germination is triggered by SLs exuded into the soil by the roots of nearby host species (Conn *et al.*, 2015; Toh *et al.*, 2015; Tsuchiya *et al.*, 2015).

Differently from other plant hormone receptors belonging to the α,β-hydrolases superfamily, SL receptors are unusual in that they are enzymatically active and able to cleave their own substrate. Although no detailed deductions about the nature, conformation, or binding mode of the D14 ligand can be made with any confidence at this stage (Carlsson *et al.*, 2018), X-ray analysis (Yao *et al.*, 2016) and enzymatic assays with pro-fluorescent probes (de Saint Germain *et al.*, 2016) suggest that as a consequence of the hydrolytic reaction catalysed by AtD14, the D-ring fragment might be trapped in the catalytic pocket; previous *in silico* modelling analyses had indeed predicted that the hydrolysis product would dock in D14 better than the intact SL, hence causing catalysis to stall (Gaiji *et al.*, 2012). This prediction is consistent with the very low or null rate of catalytic turnover by D14 enzymes (Hamiaux *et al.*, 2012; de Saint Germain *et al.*, 2016) The steps described above would lead to conformational changes that would induce the downstream cascade of events and the final degradation of the receptor itself, via the proteasome pathway (Chevalier *et al.*, 2014; Zhao *et al.*, 2015; Yao *et al.*, 2016).

**Fig. 1.**
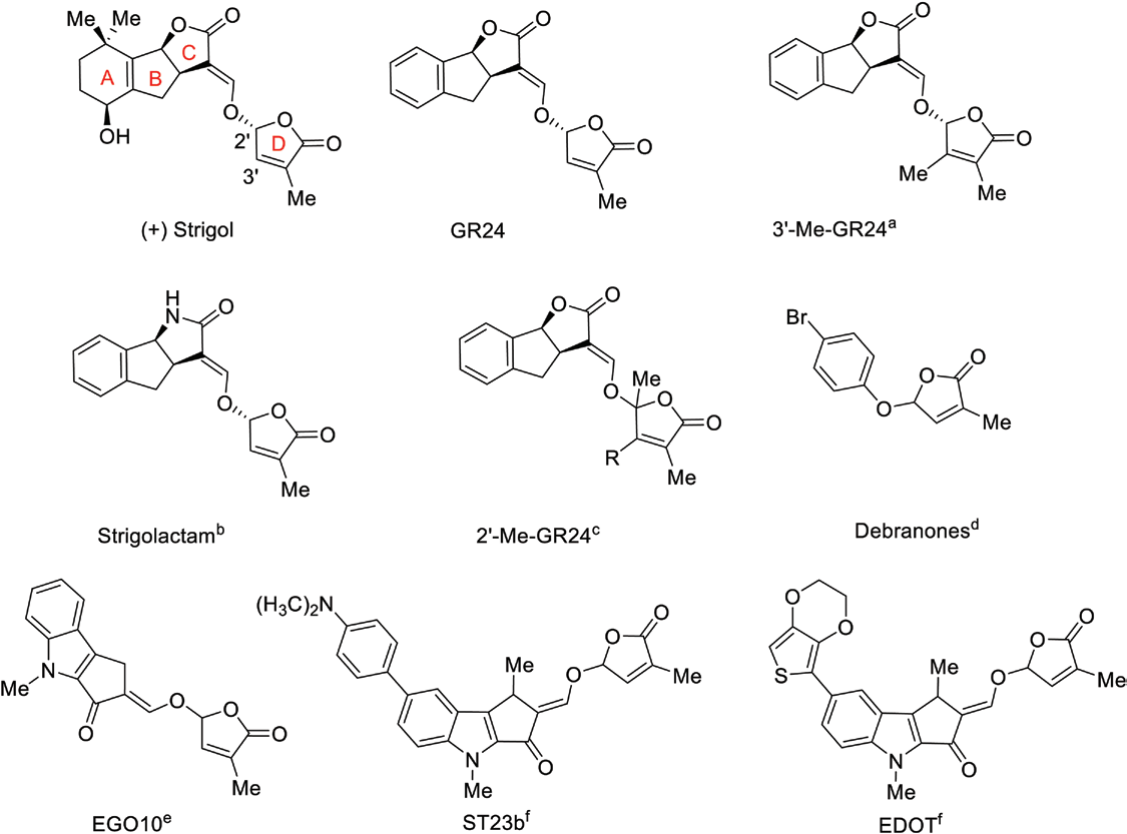
Diversity of natural and synthetic SL structures. (+) Strigol, (+)-GR24, 3′-Me-GR24, Strigolactams, 2′-Me-GR24, debranones, indolyl-series EGO. References: ^a^Boyer *et al.* (2012), ^b^Lachia *et al.* (2015*a*), ^c^Mwakaboko and Zwanenburg (2016), ^d^Fukui *et al.* (2011), ^e,f^Prandi *et al.* (2011).

Based on the above, it is evident that the D-ring is a key player in the molecular mechanism that forms the basis of SL-induced effects in plants. The relationship between bioisosteres, substituents, or groups with similar physical or chemical properties that impart similar biological properties to a chemical compound is called bioisosterism. Application of its principles (Lolli *et al.*, 2015) to such a key SL moiety as the D-ring represents a potentially valuable tool to elucidate the molecular events that happen within the active pocket during the physical interaction between receptor and ligand, and at the same time may lead to the identification of new agonists or antagonists of SLs. Among the reported synthetic analogues with modifications at the butenolide D-ring, most carry additional substituents at the 3′ (Boyer *et al.*, 2012, 2014) or 2′ positions (Mwakaboko and Zwanenburg, 2016) (Fig. 1). Based on the assumption that modifications of the functional groups in the D-ring may affect the activity of SL derivatives, and on the application of bioisosterism principles, we became intrigued by the possibility of gaining further insight into the mechanism of action of SLs by selectively modifying the reactivity of the D-ring via replacement of the butenolide with a lactam functional group. Analogues of SLs with a lactam C-ring have recently been described (Lachia *et al.*, 2014, 2015*a*, 2015*b*; Lumbroso *et al.*, 2016) and they were found to retain good activity with regards to seed germination of the parasitic weed *Orobanche cumana*. Compounds with different combinations of C-, D-lactam modifications have recently been patented by Syngenta (Lachia *et al.*, 2014, 2015*b*). We therefore designed and synthesized D-lactam derivatives of SL analogues and mimics (hereafter called SL-D-lactams) (Lombardi *et al.*, 2017) and evaluated their biological activity.

Structure–activity relationship (SAR) studies of new SL analogues must be based on homogeneous datasets containing quantitative and comparable information on bioactivity that provide molecular/physiological output of receptor–ligand interactions. A bioassay to provide such data is currently unavailable for the hormonal function of SLs, for which SAR studies currently rely either on barely quantitative and low-throughput assays on inhibition of axillary bud outgrowth (de Saint Germain *et al.*, 2016) or on *in vitro* biochemical assays such as thermal destabilization of the D14 recombinant protein (Hamiaux *et al.*, 2012), or on heterologous tests such as the promotion of physical interaction between D14 and its partner, the F-box protein MAX2 (MORE AXILLARY GROWTH2), in yeast-two-hybrid assays (Toh *et al.*, 2014). It has only been recently that a genetically encoded biosensor named StrigoQuant was devised for quantifying SL activity and specificity (Samodelov *et al.*, 2016).

Hence, in this study, we evaluated the docking in D14 and the biological activity of newly synthesized SL-D-lactams in order to investigate whether the different reactivity of lactones versus lactams towards nucleophiles would lead to differential interaction with, and possibly modification by, the D14 receptor, so to induce diverse biological outputs. To this end, we also implemented a novel bioassay based on D14 destabilization triggered by SL perception (Chevalier *et al.*, 2014). We generated transgenic Arabidopsis lines expressing AtD14 fused to firefly luciferase (LUC) for use as luminescent read-outs for quantitative measurements of SL activity, the rationale being that luminescence will be quenched by D14::LUC degradation after hormone binding. After proof-of-concept and assay calibration with one natural and several synthetic SL analogues, we performed SAR studies on a novel set of lactam derivatives using this *in planta* assay. The results obtained were compared with those of canonical germination assays on *Phelipanche aegyptiaca* seeds, and rationalized by a dedicated *in silico* study aimed at describing the binding modes of the synthesized compounds to the receptor of SLs in plants.

## Materials and methods

### Synthesis

Synthetic procedures, characterization, and absolute configuration assignments are reported in Lombardi *et al.* (2017).

### Stability

Aqueous solutions of the compounds to be tested (200 μg ml^–1^) were incubated at 25 °C in HPLC vials. The compounds were first dissolved in methanol (30%) or acetonitrile (50%) and then diluted to the final concentrations with water. The time-course of degradation was monitored by HPLC using an Agilent Technologies HPLC chromatograph 1200 Series equipped with a photo-diode array (PDA) detector, a binary-gradient high-pressure pump, and an automatic sampler. The column used was a LiChroCART® 125-4 LiChrospher® 100 RP-18 (5 μm, Merck Millipore) maintained at 25 °C. The solvents were (A) water + 0.1% formic acid and (B) acetonitrile, and the flow rate was 0.8 ml min^–1^. The initial mobile phase, 95% A / 5% B, was held for 3 min and then ramped linearly to 100% B at 23 min and held for 5 min before resetting to the original conditions. The sample injection volume was 10 μl. PDA detection was by absorbance in the 200–600-nm wavelength range. Peak detection was at the optimum wavelength (254 nm) and peak areas were used for quantification. Initial and subsequent measurements of peak area attributable to the tested compound were used to fit exponential half-life curves and to calculate first-order rate constants. Stability data allowed for calculation of the time in hours for half of the tested compound to be hydrolysed (*t*_1/2_).

### Germination activity

Seeds of *Phelipanche aegyptiaca* were collected from field-grown tomato in the West Galilee region of Israel. The seeds were stored in glass vials in the dark at room temperature until their use in germination tests. For the preparation of test solutions, the compound to be tested was weighed out very accurately, dissolved in acetone at 10^–2^ M and then diluted with sterile distilled water to the desired concentrations. All solutions were prepared just before use. Seeds were surface-sterilized and preconditioned as described by Bhattacharya *et al.* (2009). Briefly, after exposure for 5 min to 50% (v/v) aqueous solutions of commercial bleach (2% hypochlorite), seeds were rinsed with sterile distilled water. For preconditioning, seeds were placed on glass fibre filter discs using a sterile toothpick (approximately 50 seeds per disc); the glass fibre discs were placed on two filter paper discs, wetted with sterile distilled water, and incubated at 25 °C in the dark for 6 d. The preconditioned seeds were then allowed to dry completely in a laminar flow cabinet, after which they were treated with each compound at five different concentrations: 10^–5^ M, 10^–6^ M, 10^–7^ M, 10^–8^ M, and 10^–9^ M. Their germination rate was evaluated under a stereomicroscope 7 d after the beginning of the treatment. For each concentration, at least 250 seeds were scored; synthetic SL *rac*-GR24 was included as a positive control across the same range of concentrations, while a solution of 0.001% acetone in sterile distilled water was included as a negative control. Seeds were scored as germinated if the radicle protruded through the seed coat 1 week after treatment. Germination values were normalized to those of *rac*-GR24 at 10^–7^ M.

### Luminometer assays

**Fig. 2.**
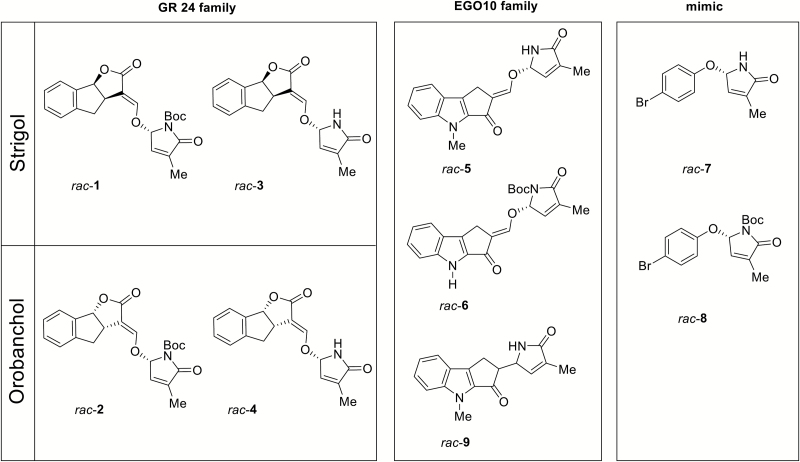
Group of D-lactam SL analogues and mimics used in this study. *Rac*-**1** and *rac*-**2** are the *N*-Boc-protected GR24 D-lactam diastereoisomers. *Rac*-**3** and *rac*-**4** are the NH GR24 D-lactam diastereoisomers. *Rac*-**5** and *rac*-**6** are NH and *N*-Boc D-lactam EGO10 derivatives, respectively (Prandi *et al.*, 2011). *Rac*-**7** and *rac*-**8** are mimic D-lactams for NH and *N*-Boc, respectively (Fukui *et al.*, 2011). *Rac*-**9** is a EGO10 derivative lacking the enol ether bridge.

A binary D14p::D14::LUC vector was obtained by LR-recombination (Invitrogen) of a pDONOR207 carrying the D14 promoter fused to the D14 CDS (Chevalier *et al.*, 2014) in pGWB435 (Nakagawa *et al.*, 2007). Transgenic D14::D14::LUC Arabidopsis plants were generated by agroinfiltration of Col-0 plants using the floral dip method (Clough and Bent, 1998). Seeds were surface-sterilized with an 8% aqueous solution of commercial bleach in distilled water for 5 min and rinsed five times with sterile distilled water. Sterile seeds were plated on MS medium (pH 5.8) (Murashige and Skoog, 1962) without sucrose solidified with 1.2% Agar, and kept at 4 °C for 3 d (stratification). Seeds were then incubated for 7 d in a growth chamber at 25 °C and a photoperiod of 16 h light/8 h dark. SL analogues and mimics (Figs 1, 2) were accurately weighed and dissolved in acetone at 10^–2^ M. Five different concentrations (10^–4^ M, 10^–5^ M, 10^–6^ M, 10^–7^ M, 10^–8^ M) were prepared by 1:10 serial dilutions in liquid MS medium, together with blank controls containing corresponding water and acetone volumes in the medium. D-Luciferin (potassium salt) stock was prepared at 25 mg ml^–1^ in DMSO, aliquoted and stored at –80 °C until use; all other solutions were prepared just before the assay. Using tweezers, 7-d-old Arabidopsis seedlings were placed in a 96-well microtiter plate with their cotyledons facing up (one seedling per well, in 170 µl of liquid MS medium), and 15 µl of luciferin (0.125 mg ml^–1^, diluted 1:200 in MS from stock) was added to each well, corresponding to 1.875 µg. The plate was covered with a transparent film with a hole poked above each well to allow for gas exchange. Measurements were taken every 15 min in a multimode reader (LB942 Tristar2 S, Berthold Technologies) for the following 24 h; the signal was allowed to stabilize for 2–3 h in the light before treatments were started by adding 15 µl per well of the different SLs in MS medium, thus reaching a final volume in each well of 200 µl and 9.4 µg ml^–^ of luciferin. Appropriate blank controls were added as well. Each treatment was applied to a minimum of 16 wells (seedlings), each of which was measured individually over time. The percentage efficacy of each compound molecule was calculated 6 h after treatment as a function of the decrease in D14::LUC-emitted luminescence with respect to (+)-GR24 1 µM (i.e. GR24^5DS^), assuming that the latter, minus the drift of the corresponding blank control, had 100% efficacy. For calibration purposes, SL analogues that had already been characterized, namely (±)-ST23b, (±)-EGO10, and (±)-EDOT (Prandi *et al.*, 2011), were tested across the same concentration range, along with (±)-strigol (Chiralix, NL). The half maximal-effective concentration (EC_50_) for (+)-GR24 was calculated by linear regression fitting of the data (*n*=5, with at least three individual seedlings and values being pooled for each replicate) at the different concentrations, minus the values for acetone-treated samples (negative controls) and normalized in relation to (+)-GR24 0.01 μM, which was set to 0%. Confidence intervals at 95% were used to express errors of the means.

### Docking models

All novel compounds were docked in the protein binding site using the docking software GOLD, version 5.5 (www.ccdc.cam.ac.uk). For each compound, 25 diverse poses were generated and analysed. A radius of 10 Å was used to define the pocket extension. Automatic default parameters were set for the Genetic Algorithm. Shape constraints were imposed using as template the structure of GR24 co-crystallized within the target (PDB code 5dj5; Zhao *et al.*, 2015). ChemScore was used as the scoring function. All calculations were performed on a Dell Precision workstation, having two Intel Xeon processors, twelve core 1TB 7.2K 6GBPS SAS Hard Drive, NVidia GTX 980 graphic card, and a Linux operating system centos 7, kernel version 3.10.0-514.10.2.el7.x86_64. Molecular interaction fields were calculated using FLAP (Fingerprints for Ligands and Proteins, Baroni *et al.*, 2007; Grossert *et al.*, 2015), using the DRY probe to describe potential hydrophobic interactions, and the sp2 carbonyl oxygen O and the amide N1 probes for hydrogen-bond donor and acceptor regions, respectively.

## Results

### Molecules

A range of molecules was considered and used for bioassays, with (+)-GR24 used as the reference compound. Strigol was included as being representative of natural SLs. ST23b, EGO10, and EDOT were selected for their reported high activity in inducing germination and hyphal branching in *P. aegyptiaca* seeds and the AM fungus *Gigaspora margarita*, respectively, along with their ability to affect root architecture (Prandi *et al.*, 2011; Cohen *et al.*, 2013; Mayzlish-Gati *et al.*, 2015). SL analogues in the form of D-lactam derivatives were synthesized according to Lombardi *et al.* (2017). We decided to synthesize *N*-Boc-protected derivatives of GR24-D-lactam as a racemic couple of diasteroisomers (*rac*-**1** and *rac*-**2**, Fig. 2) based on the assumption that the presence of the encumbering Boc group may affect molecular accommodation in the receptor, both in terms of space and of H-bonding interactions with catalytically important amino acids of the receptor pocket. The same series of compounds was obtained as *N*-unprotected derivatives, *rac*-**3** and *rac*-**4**. The stereochemistry of *rac*-**1** and *rac*-**3** corresponded to the strigol family (Lombardi, 2017), while that of *rac*-**2** and *rac*-**4** corresponded to the orobanchol family of natural SLs. *Rac*-**5** and its *N*-Boc precursor *rac*-**6**, based on the EGO10 backbone (Fig. 2), were also synthesized and used for further investigations.

In order to explore the effect of a lactone-to-lactam modification in the SL-mimics category, we also synthesized two SL mimics, *rac*-**7** and *rac*-**8**, as NH D-lactam and *N*-Boc precursor, respectively (Fig. 2). In addition, we designed and synthesized a compound (*rac*-**9**, Fig. 2) whose structure was strictly related to EGO10-D-lactams but lacked the enol ether bridge connecting the ABC core to the D-ring. In *rac*-**9**, these latter two parts were instead directly linked together. In terms of bioactivity, this meant that, in principle, the molecule could accommodate into the receptor pocket but could not be hydrolysed in the way that SLs are, according to the current understanding of receptor–ligand interactions.

### Stability

Natural and synthetic SLs are rather sensitive to hydrolysis at pH 7 and are readily decomposed through the cleavage of the D-ring at pH 9.38 (Vurro *et al.*, 2016). Therefore, differences in activity among SL analogues may be attributed to their instability in the aqueous medium. In order to address this point, the stability in aqueous solutions of the newly synthesized SL-D-lactams was tested, and compared to the (+)-GR24 standard (Kannan and Zwanenburg, 2014; Halouzka *et al.*, 2018). Two different conditions were considered, a 30% solution of MeOH in water and a 1:1 solution of acetonitrile in water. As expected, stability in MeOH was highly compromised for all compounds, but to a greater extent for analogues showing both the Michael acceptor function (enol ether bridge) and an unprotected N in the D-lactam ring, as for *rac*-**3**, *rac*-**4**, *rac*-**5**, and *rac*-**7** (Table 1): after a few hours 50% of the compounds were degraded. This was not surprising from a chemical point of view, as the functional group in SL-D-lactams is an aminal, which is more prone to hydrolysis than the acetal of the natural SL skeleton. By contrast, *rac*-**9**, in which the enol ether bridge was missing and the lactone C-ring was directly connected to the lactam D-ring, showed high stability in both solvents. All compounds with an *N*-Boc-protected function showed higher stability (*rac*-**1**, *rac*-**2**, *rac*-**6**, *rac*-**8**) compared to their unprotected (NH) versions. For the GR24-family compounds, both NH (*rac*-**3**) and *N*-Boc (*rac*-**4**) lactams showed very low stability values, as the half-life time was estimated to be around 3 h. For *rac*-**5**, the NH compound in the EGO10 family, the half-life dropped to 2 h. These data should be taken into account when considering the results of both bioassays (parasitic seed germination and D14 degradation tests).

**Table 1.** Chemical stability of lactams, named as described in Fig. 2, in 30% MeOH or 1:1 acetonitrile (ACN): water at 21 °C and pH 6.7.

Compound	Half-life (*t*_1/2_, h)*	
	30% MeOH	ACN:Water, 1:1
(+)-GR24	80	3375
*rac*-**1**	110	720
*rac*-**2**	21	140
*rac*-**3**	3	3.8
*rac*-**4**	3.3	4.4
*rac*-**5**	2	24
*rac*-**6**	190	528
*rac*-**7**	11	17
*rac*-**8**	230	1080
*rac*-**9**	1100	2900

* *t*_1/2_ values were extrapolated from the plots of peak area versus time.

### Germinating activity

**Fig. 3.**
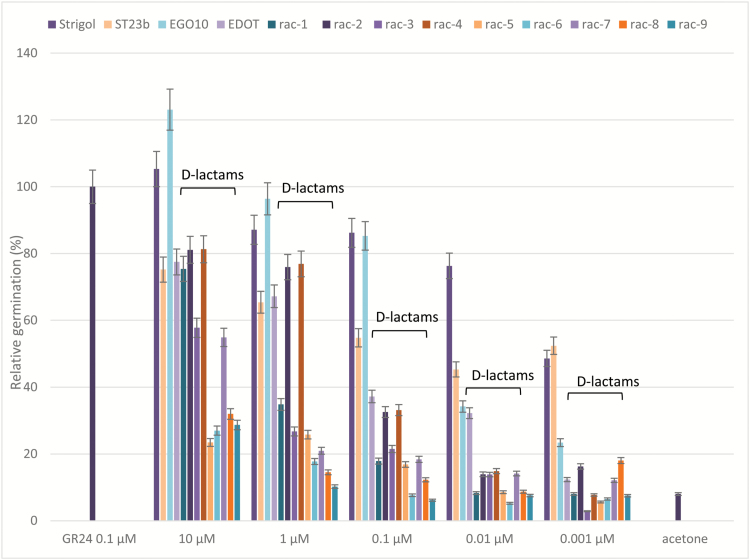
Germination-inducing activity on *Phelipanche aegyptiaca* seeds of *rac*-**1**–**9** and of strigol, ST23b, EGO10, and EDOT at different concentrations, compared to *rac*-GR24 0.1 μM as a positive control and to acetone as a negative control. The *y*-axis shows the percentage of germinated seeds normalized to *rac*-GR24 at 0.1 μM, which was set to 100%. The *x*-axis indicates the range of concentrations. Data are means (±SD) of *n*>250 seeds. Confidence intervals at 95% are used to express errors of the means. The data for for *rac*-**1**–**9** are indicated by ‘D-lactams’.

The newly synthesized SL-D-lactams were assayed on seeds of *P. aegyptiaca* and compared to *rac*-GR24 as the reference standard, and to strigol and to the analogues ST23b, EGO10, and EDOT (Prandi *et al.*, 2011) (Fig. 3).

With respect to *rac*-GR24, the maximum activity of which was recorded at concentrations equal to or above 0.1 μM (Fig. 3 and data not shown), the dose–response curves of strigol, ST23b, EGO10, and EDOT corresponded to those already reported in the literature (Prandi *et al.*, 2011). The D-lactams *rac*-**1**–**9** were all less effective in comparison with (+)-GR24: *rac*-**1**, **3**, and **7** showed high activity only at concentrations equal to 10 μM, thus indicating ~100-fold lower potency than *rac*-GR24. The GR24-D-lactams *rac*-**2** and *rac*-**4** (orobanchol family, Fig. 2) were the most active compounds of the series, as some germination activity could be detected even at 1 μM; all other compounds were inactive throughout the whole range of concentrations. Surprisingly, *rac*-**2** and **4** showed overlapping activity profiles, as if the presence of the Boc group on *N* was not affecting the perception by parasitic seeds. The same trend could be observed for all other compounds, for which a substantial difference between cognate NH and *N*-Boc derivatives could not be detected.

### Luminometer assays

**Fig. 4.**
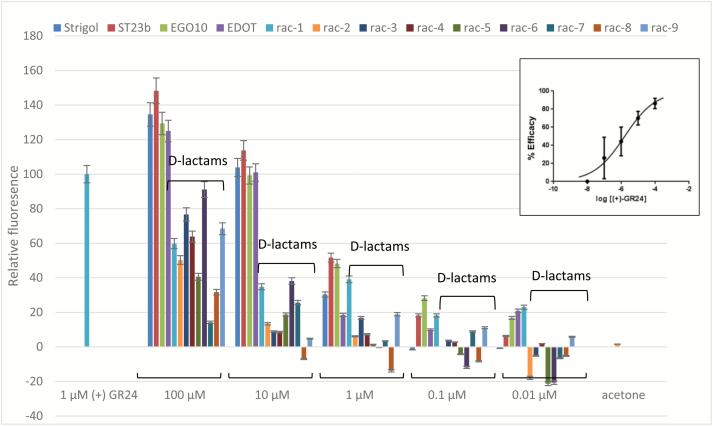
Luciferase assay. The assay was calibrated using (+)-GR24 across a range of concentrations (0.01–100 μM). Data are means (±SD) of *n*=5 replicates, where each replicate consists of at least three pooled individual seedlings and readings. The data are normalized to GR24 at 0.1 μM, which was set to 100%. The data for *rac*-**1**–**9** are indicated by ‘D-lactams’. Inset: The EC_50_ curve for (+)-GR24 obtained using GraphPad Prism 7.00. The curve was calculated by linear regression fitting of the data (*n*=5, where each replicate consisted of at least three pooled individual seedlings and readings) at different concentrations, minus values for acetone-treated samples (negative controls) and normalized to GR24 at 0.01 μM, which was set to 0%. Confidence intervals at 95% are used to express errors of the means.

D14 is a target for proteasome-dependent destruction upon interaction with its ligand(s), which explains why fluorescence of D14::GFP fusion proteins is quenched upon SL treatment in transgenic Arabidopsis (Chevalier *et al.*, 2014). We exploited this molecular network to implement a quantitative activity assay that inversely correlated luminescence to perception of SL-related molecules in transgenic Arabidopsis expressing D14::LUC under the control of the D14 endogenous promoter. We calibrated the assay using (+)-GR24 over a range of concentrations (Fig. 4, inset), and the calculated EC_50_ value was 1.62 μM (see Supplementary Table S1 at *JXB* online). We then used the assay to test various SLs in the same range, namely strigol as an example of natural SLs; ST23b, EGO10, and EDOT (Fig. 1, all used as *rac* mixtures) as examples of active SL analogues; and the new set of SL-D-lactams *rac*-**1**–**9** (Fig. 2). As shown in Fig. 4, strigol, ST23b, EGO10, and EDOT induced high levels of D14 degradation [albeit though less efficiently than the pure enantiomer (+)-GR24], whilst for the compounds of the D-lactam series some activity was detectable only in the 10–100 μM range.

**Fig. 5.**
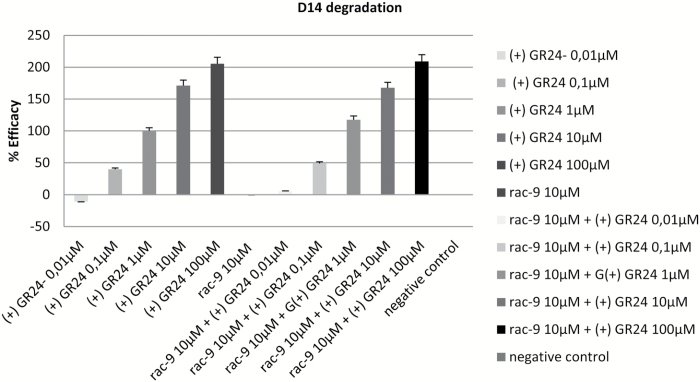
Luciferase competition test between *rac*-**9** and (+)-GR24. (A) Efficacy values for (+)-GR24 across a range of concentrations, normalized to the value at 1 μM, which was set to 100%. (B) Efficacy values for (+)-GR24 across the same range of concentrations as in (A), but in the presence of *rac*-**9** at 10 μM. Data are means (±SD) of *n*=5 replicates, where each replicate consisted of at least three pooled individual seedlings and readings.

As *rac*-**9** was inactive in both bioassays at 10 µM, we then tested whether it could behave as an antagonist in a luminometer-based competition assay. For this purpose, *rac*-**9** was kept constant at the highest inactive concentration (10 µM), while concentrations of (+)-GR24 were varied in the range 0.01–100 µM. The efficacy values in these samples in terms of luminescence quenching were compared with those of samples receiving (+)-GR24 alone (positive control). As shown in Fig. 5, the results indicated no antagonistic behaviour for *rac*-**9** under our experimental conditions.

### Docking studies

**Fig. 6.**
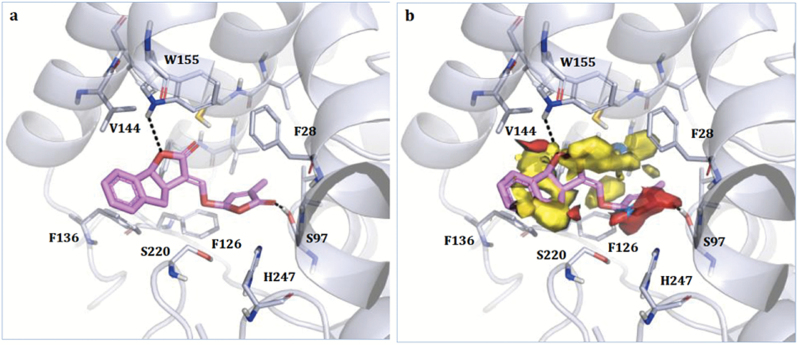
(A) Crystallographic pose of (+)-GR24 within rice D14 (PDB code 5dj5; Zhao *et al.*, 2015). The ligand and the residues lining the pocket are shown as coloured regions. Hydrogen bonds are represented as black dashed lines. Residues 158–166 have been removed for clarity, and only the residues closest to the ligand are labelled. (B) Molecular Interaction Fields for the enzyme pocket calculated using FLAP (Baroni *et al.*, 2007; Spyrakis *et al.*, 2015). Red, blue, and yellow indicate the hydrogen-bond acceptor, hydrogen-bond donor, and hydrophobic Molecular Interaction Fields, respectively.

In order to interpret the activity data of GR24-based lactams in light of their possible binding mode within the SL receptor, we performed docking simulations for three SL-D-lactam compounds within the binding pocket of D14. We selected as a template the structure of rice D14 co-crystallized with GR24 (PDB code 5dj5; Zhao *et al.*, 2015), given its high similarity to the ligands under study and the high conservation of the binding-site residues. As previously noted, the docking pose showed only one enantiomer of GR24 in the pocket, thus suggesting it to be the most probable substrate. The X-ray structure of the complex is shown in Fig. 6A. The compound is H-bonded to the catalytic Ser97 and to Trp155. The correspondence of the hydrophobic and H-bond-donor moieties of the ligand with the same GRID Molecular Interaction Fields of the pocket is shown in Fig. 6B. The yellow contours correspond to hydrophobic areas while the red and blue regions identify hydrogen bond acceptor and donor areas, respectively.

**Fig. 7.**
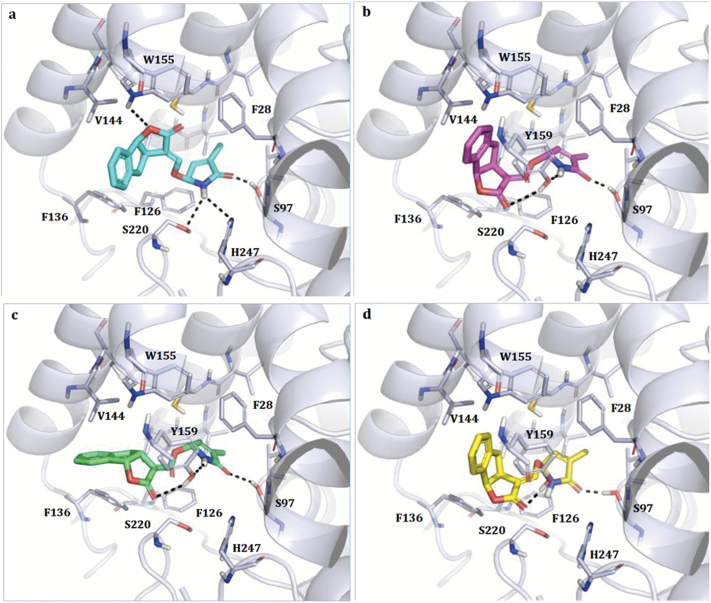
Docking models of *rac*-**3** and *rac*-**4** in the binding site of rice D14. For each racemic mixture, both enantiomers were modelled. (A) *Rac*-**3**, (SSR, strigol configuration), (B) *rac*-**3** (RRS *ent*-strigol configuration), (C) *rac*-**4** (RRR, orobanchol configuration), and (D) *rac*-**4** (SSS, *ent*-orobanchol configuration). The ligand and the residues lining the pocket are shown as coloured regions. Hydrogen bonds are represented as black dashed lines. The protein is represented as a simplified model. Residues 158–166 have been removed for clarity. Tyr159 is shown only when it is relevant for the stabilization of the complex. Only the residues closest to the ligand are labelled.

We used the same approach to investigate the pose of the new D-lactams *rac*-**3**, *rac*-**4**, and *rac*-**9** in the D14 pocket. *Rac*-***3*** was the GR24-D-lactam whose configuration was ‘strigol-type’ while *rac*-***4*** corresponded to the ‘orobanchol-type’. Both enantiomers for *rac*-**3** and *rac*-**4** were docked in the enzyme pocket. As expected, the most reasonable pose was obtained for the enantiomer of *rac*-**3**, which possessed absolute configuration (SSR), had the same stereochemistry as (+)GR24 co-crystallized with D14 (Fig. 7A), and showed a very similar orientation. The ligand was able to interact with the catalytic Ser97 and His247, with Ser220, and also with Trp155, lining the upper part of the binding site. Hydrophobic moieties properly fitted the pocket hydrophobic region lined by Phe28, Phe126, Phe136, and Val144. Properly located and stabilized through hydrophobic and electrostatic interactions in the binding site, the molecule could then be easily hydrolysed by Ser97 and thus mimic SL activity. A less favourable pose (RRS) was obtained for the enantiomer (*ent*-strigol configuration, Fig. 7B), which maintained the contact with Ser97 but, because of the different stereochemistry, moved the hydrophobic condensed ring towards Trp155 and lost the contact with Ser220 and His247. Additional interactions were made with Tyr159, as shown in Fig. 7B. Similar to *rac*-**3**, *rac*-**4** also showed a less reliable pose than the co-crystallized GR24, again in agreement with poor activity data in the D14::LUC degradation bioassay. In particular, both enantiomers maintained the interaction with the catalytic Ser97 and with Tyr159, and both experienced an adjustment of the pyrrolone ring and of the indeno-furan system. *Rac*-**1** and *rac*-**2** did not give any reasonable pose when docked in D14, because of the presence of the Boc group (data not shown).

**Fig. 8.**
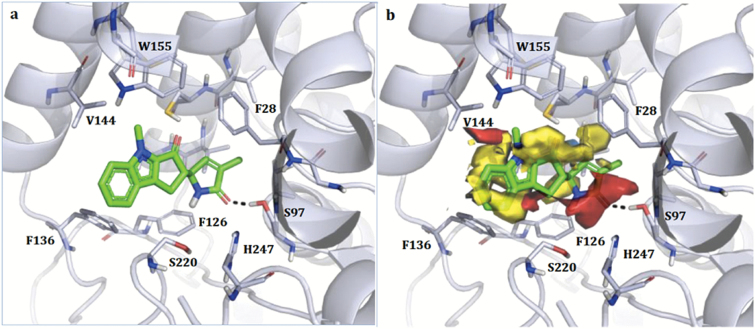
Docking models of *rac*-**9** in the binding site of rice D14. Only the SS enantiomer is shown. (A) Crystallographic pose. The ligand and the residues lining the pocket are shown as coloured regions. Hydrogen bonds are represented as black dashed lines. The protein is represented as a simplified model. Residues 158–166 have been removed for clarity, and only the residues closest to the ligand are labelled. (B) Molecular Interaction Fields as calculated using FLAP (Baroni *et al.*, 2007; Spyrakis *et al.*, 2015). Red, blue, and yellow indicate the hydrogen-bond acceptor, hydrogen-bond donor, and hydrophobic Molecular Interaction Fields, respectively.

*Rac*-**9** was also docked in D14 (Fig. 8A); only the enantiomer (SS) that gave the best pose is shown in the figure. When located in the pocket, the pyrrolone ring of *rac*-**9** maintained the interaction with Ser97 but no other H-bond was formed. Hydrophobic and polar groups both superimposed quite well with the corresponding Molecular Interaction Fields, with the exception of the indolone methyl group, which was probably too close to Trp155. Nevertheless, due to the reduced number of hydrogen bonds, the presence of negative hydrophobic–polar contacts, and the higher rigidity of the molecule, the D14 complex with *rac*-**9** was likely to be far less stable than the one with (+)-GR24.

## Discussion

The crystal structure of rice D14 in a complex with intact GR24 was first determined in 2013 through use of a high GR24 molar ratio, extensively screened crystallization conditions, and further soaking with fresh GR24 (Zhao *et al.*, 2013). However, while the protein was well resolved, the ligand had poor resolution, indicating its incomplete occupancy (Carlsson *et al.*, 2018). In the same year, the structure of the SL receptor for host-produced SLs in the hemiparasitic plant *S. hermontica* was also obtained (Toh *et al.*, 2015), and it showed a good superimposition with the rice D14 structure (root-mean square deviation of atomic positions, RMSD, equal to 0.8 Å) and a very well conserved binding site. The mode of action of SLs and the enzymatic role of the receptor, however, still remain to be completely elucidated. Biochemical assays shed light on the molecular mechanisms inside the pocket and helped in the identification of the hydrolysis products (Snowden and Janssen, 2016); however, it was also demonstrated that hydrolysis at the receptor is not mandatory to activate the cascade of events leading to SL-related effects, because even non-hydrolysable compounds could act as germination stimulants (Toh *et al.*, 2014). In spite of these uncertainties, plenty of SAR data point to the D-ring of the SL frame as being the crucial bioactiphore of this class of phytohormones. In this study, we designed a series of compounds in which the butenolide lactone D-ring was changed into a lactam, the reactivity of which towards nucleophiles is decreased compared to a lactone. After a stability survey in different solvents, the bioactivities of this set of molecules were initially tested using a germination assay on seeds of *P. aegyptiaca*. The test in itself is trivial, but it is very sensitive and widely used to obtain preliminary clues about the germination-inducing activity of new compounds. All the SL-D-lactams proved to be less potent inducers of germination than *rac*-GR24, with *rac*-**1**, *rac*-**2**, and *rac*-**4** showing the highest activity; at 10 μM SL-D-lactams were comparable to *rac*-GR24 at 0.1 μM, i.e. ~100-fold lower activity. *Rac*-**9** was inactive even at 10 μM. Surprisingly, *N*-Boc-derivatives (*rac*-**1**, *rac*-**2**, *rac*-**6**, and *rac*-**8**) were as active as the corresponding NH structures. This was unexpected as, in principle, the bulking-group Boc can barely be accommodated in the receptor pocket, as confirmed by the docking simulations. However, our results might be explained by the active pocket in the D14-like receptor(s) of the parasitic plants being larger than in D14 (Toh *et al.*, 2015), and/or with the Boc group being lost just before the molecule reaches the active site. We may then assume that the removal of the Boc group, which results in an unprotected compound, occurs at some point along the pathway that leads to the target site, and probably is due to other sources of catalysis present *in vivo* (de Groot *et al.*, 2000). To overcome these inherent uncertainties, and to obtain SAR data for the D14-dependent hormonal activity of SLs, we then implemented a novel *in planta* bioassay based on the measurement of the decrease in luminescence of transgenic Arabidopsis expressing a translational D14::LUC fusion. It has been suggested that SL-triggered D14 degradation may be needed in order to maintain the rate of signalling for D14-type receptors at a ratio of one SL molecule to one receptor, and thus that the SL signal is not amplified (at least at the level of perception) (Lumba *et al.*, 2017). This makes it possible that SAR data obtained using the LUC assay do not fully overlap with data obtained using more traditional assays of hormonal function, as downstream transduction cascades would (typically) lead to signal amplification instead. There is a further associated risk that receptor degradation and physiological activity triggered by ligand perception may be uncoupled or non-linearly coupled under certain conditions, as has been shown for MAX2-independent KAI2 degradation (Waters *et al.*, 2015). Notwithstanding this caveat (which could only be dismissed after careful comparison of the LUC bioassay output with other, more traditional bioassays and with a wider range of SL structures), the LUC assay could be initially calibrated convincingly with the pure enantiomer (+)-GR24 and it proved to be able to robustly report bioactivity across a wide dynamic range, with very good output reproducibility and a sensitivity threshold between 10^–8^ and 10^–7^ M for (+)-GR24. The EC_50_ value was in the μM range, i.e. within the range for GR24 to induce physiological responses, and within that commonly adopted for exogenous treatments. In a further step, together with strigol, a small set of SL analogues known to be active in a number of other bioassays were used to successfully confirm the soundness and sensitivity range of the assay. The cognate test StrigoQuant (Samodelov *et al.*, 2016) is more sensitive. This is most likely due to the fact that in StrigoQuant the reporter construct is expressed under the control of a strong constitutive promoter instead of the D14 endogenous one, that the SMXL6 (SUPPRESSOR OF SMAX1-LIKE6) reporter is a direct target of MAX2 and is thus more quickly degraded upon SL perception than D14 itself, and that treatments are delivered to protoplasts rather than to intact plants, which will need to absorb the compounds being tested through their roots and to translocate them systemically before a signal can start to be recorded (taking several minutes for very active compounds in the micromolar range). However, for the same reasons, the test reported here is less laborious, expensive, and technically demanding than StrigoQuant; it is also a true whole-plant bioassay in which the (reporter-tagged) receptor is expressed according to its native physiological level and profile.

Once it was established that the assay was suitable for SAR studies, it was used to evaluate the activity of our novel group of synthetic SL-D-lactam derivatives, and the results were compared with those of the more canonical germination assay of seeds of parasitic plants. All the compounds belonging to the lactam series proved to be inactive at concentrations equal to or lower than 10 μM, while weak activity was detected only at 100 μM. *Rac*-**9**, the non-hydrolysable compound of the series, showed the same activity profile as the other members of the SL-D-lactam class, and was substantially inactive. By contrast, the D-lactone series members strigol, ST23b, EGO10, and EDOT responded well to the test conditions, inducing a regular decrease of luminescence intensity for decreasing concentrations. At 1 μM they showed significant activity, albeit weaker than (+)-GR24. In this regard, it should be noted that all the synthetic analogues as well as strigol were used as racemic mixtures, while the reference was the pure (+)-GR24 enantiomer.

To assess whether the lack of activity of the D-lactam compounds was exclusively attributable to the reduced reactivity of the D-lactam versus the D-lactone ring, or whether it was the result of poor accommodation of the molecule into the receptor pocket, docking simulations were undertaken for *rac*-**3**, *rac*-**4**, and *rac*-**9**. The results showed that, albeit with slight differences, NH derivatives of the SL-D-lactams series could dock favourably in the receptor pocket, while the *N*-Boc derivatives could not. This finding supports our contention that in order for the germination and D14::LUC degradation data to be explained, the Boc group must be lost before the ligands reach the catalytic pockets. On the other hand, the fact that *rac*-**3** was almost inactive can be explained by the high intrinsic instability of the compound, the half-life time of which (<4 h) is shorter than the measurement time, independent of the bioassay.

Similarly, *rac*-**4** also showed less reliable poses in D14 than the co-crystallized GR24, again in agreement with its weak activity in the luciferase bioassay. As a germination inducer, however, *rac*-**4** at 1 μM could attain an efficiency comparable to GR24, even if its potency was ~10-fold lower, which was possibly because of its instability. The enhanced sensitivity towards SLs and their analogues in the parasitic versus producing host plants (in the picomolar versus micromolar range) (Toh *et al.*, 2015) could possibly explain this apparent discrepancy.

Among the D-lactams, *rac*-**9** was designed to resist hydrolysis and this was confirmed by the high stability of the compound in strong nucleophilic solvents (*t*_1/2_ in the range 1000–3000 h, depending on the solvent). Due to its having very little activity in both the bioassays used, we initially suspected that *rac*-**9** was possibly acting as a SL antagonist. However, a competition experiment with (+)-GR24 at various concentrations indicated that it did not possess antagonistic activity, at least under our experimental conditions, although at very high concentration (100 μM) it behaved as a partial agonist. The docking results for this compound indicated, as a possible explanation, that the *rac*-**9**–D14 complex could be not stable enough for *rac*-**9** to act as a competitive inhibitor of (+)-GR24.

## Conclusions

In this work, we have presented a novel *in planta* bioassay, which although more indirect than a biochemical interaction assay, conveys a biologically meaningful output, has an acceptable dynamic range, is relatively simple to execute, is up-scalable, and is robust enough to be exploitable for SAR studies. We employed this test to evaluate the biological activity of a class of novel SL analogues in which the lactone on the D-ring was changed into a lactam. SL-D-lactams showed much weaker activities than canonical SL-D-lactones. Docking studies demonstrated that these molecules fitted perfectly into the D14 pocket, establishing almost the same interactions with the catalytic triad as active SLs. Assuming that the mode of action of SLs relies on a nucleophilic reaction occurring inside the receptor onto the butenolide D-ring, the reasons for inactivity of SL-D-lactams can be then ascribed to the change of the lactone functional group to a lactam, and to the lower reactivity of the latter to nucleophiles. Alternatively, or in combination, this structural variation may affect uptake and transport of SL-D-lactams in living tissues.

## Supplementary data

Supplementary data are available at *JXB* online.

Table S1. Calibration of the D14::Luc bioassay using (+)-GR24.

Supplementary Data
