# Supplementary material for: Structure–activity relationships of strigolactones via a novel, quantitative in planta bioassay
Source: J Exp Bot. 2018 Mar 15;69(9):2333–43. doi: 10.1093/jxb/ery092 (PMC5913603; doi:10.1093/jxb/ery092)
Supplement: Supplementary Data [file ery092_suppl_supplementary_data.pdf]

Structure-activity relationship of strigolactones *via* a novel, quantitative *in planta* bioassay

Emma Artuso, Elena Sanchez, Chiara Lombardi, Ivan Visentin, Beatrice Lace, Wajeeha Saeed, Marco Lolli, Piermichele Kobauri, Zahid Ali, Francesca Spyrakis, Pilar Cubas, Francesca Cardinale, and Cristina Prandi

Supplementary data

Table S1. Calibration of the D14::Luc bioassay using (+)-GR24.

D14::Luc bioassay

(+)-GR24 calibration

GR24 % EFFICIENCY NORMALIZED WITH Low Acetone Control

	Best-fit values
LOGIC50	-5.791
HILLSLOPE	0.4764
EC_{50}	1.619e-006
Std. Error	
LOGIC50	0.1276
HILLSLOPE	0.06399
95% Confidence Intervals	
LOGIC50	-6.052 to -5.529
HILLSLOPE	0.3454 to 0.6075
EC_{50}	8.867e-007 to 2.955e-006
Goodness of Fit	
Degrees of Freedom	28
R^2	0.8498
Absolute Sum of Squares	4904
Sy.x	13.23
Number of points	
Analyzed	30
